# Screening and Expression of a Silicon Transporter Gene* (Lsi1)* in Wild-Type Indica Rice Cultivars

**DOI:** 10.1155/2017/9064129

**Published:** 2017-01-16

**Authors:** Mahbod Sahebi, Mohamed M. Hanafi, M. Y. Rafii, Parisa Azizi, Rambod Abiri, Nahid Kalhori, Narges Atabaki

**Affiliations:** ^1^Laboratory of Climate-Smart Food Crop Production, Institute of Tropical Agriculture and Food Security, Universiti Putra Malaysia, 43400 Serdang, Selangor, Malaysia; ^2^Department of Land Management, Faculty of Agriculture, Universiti Putra Malaysia, 43400 Serdang, Selangor, Malaysia; ^3^Laboratory of Plantation Science and Technology, Institute of Plantation Studies, Universiti Putra Malaysia, 43400 Serdang, Selangor, Malaysia; ^4^Department of Crop Science, Faculty of Agriculture, Universiti Putra Malaysia, 43400 Serdang, Selangor, Malaysia; ^5^Department of Biochemistry, Faculty of Biotechnology and Biomolecular Sciences, Universiti Putra Malaysia, 43400 Serdang, Selangor, Malaysia; ^6^Department of Biology, Faculty of Science, Universiti Putra Malaysia, 43400 Serdang, Selangor, Malaysia; ^7^IAU of Tehran Science and Research Branch, Tehran, Iran

## Abstract

Silicon (Si) is one of the most prevalent elements in the soil. It is beneficial for plant growth and development, and it contributes to plant defense against different stresses. The* Lsi1* gene encodes a Si transporter that was identified in a mutant Japonica rice variety. This gene was not identified in fourteen Malaysian rice varieties during screening. Then, a mutant version of* Lsi1* was substituted for the native version in the three most common Malaysian rice varieties, MR219, MR220, and MR276, to evaluate the function of the transgene. Real-time PCR was used to explore the differential expression of* Lsi1* in the three transgenic rice varieties. Silicon concentrations in the roots and leaves of transgenic plants were significantly higher than in wild-type plants. Transgenic varieties showed significant increases in the activities of the enzymes SOD, POD, APX, and CAT; photosynthesis; and chlorophyll content; however, the highest chlorophyll A and B levels were observed in transgenic MR276. Transgenic varieties have shown a stronger root and leaf structure, as well as hairier roots, compared to the wild-type plants. This suggests that* Lsi1* plays a key role in rice, increasing the absorption and accumulation of Si, then alters antioxidant activities, and improves morphological properties.

## 1. Introduction

Rice (*Oryza sativa* L.) is one of the most important food crops worldwide, and it is cultivated in a particular type of artificial wetland. Due to population increases worldwide, rice production is expected to increase by over 40% by 2030. Hence, the production of different rice varieties with greater yield stability is essential for overcoming reductions in grain yield as well as the limitations of arable lands [1–3]. Silicon (Si) is one of the most plentiful macroelements in the soil and performs an important function in healing plants under various environmental stresses. Si is applied to plants to induce resistance against various stresses, including both disease and pests. Moreover, Si can improve the condition of the soil, which may contain toxic levels of heavy metals along with other chemical elements [4]. Silicon among all plant nutrient elements inside the soil carries a distinguished role in plant formation especially under harsh environment and poor nutrient condition. The role of Si is not limited to plant growth only, but also it helps plant to cope with variety of abiotic and biotic stresses [4, 5].

Many insect pests and epidemic diseases affect different parts of the rice plant, resulting in severe rice production losses [6]. Rice yield stability is affected by various biotic and abiotic stresses. Blast and sheath blight fungi, bacterial blight, root-knot (caused by nematodes), and rice yellow mottle virus cause the majority of losses in the annual rice grain yield [7]. The brown plant hopper (BPH,* Nilaparvata lugens*), an important insect pest of cultivated rice, has interfered with the production of rice since the 1960s [8]. The plant cell wall is a barrier between host plants and pathogens, but the fungus* Magnaporthe oryzae* uses 30 and 44 different enzymes to degrade cellulose and hemicellulose, respectively, in plant cell walls [9, 10].

On the other hand, the release of chlorofluorocarbon gases due to the increased solar ultraviolet-B radiation on the surface of the earth has reduced the stratospheric ozone (O_3_) layer [11]. Depletion of the O_3_ layer decreases plant height and aboveground biomass, and it also increases DNA damage. In this context, it has been shown that increased UV-B radiation has the same effect on plants grown in the Arctic, Antarctic, and lower latitudes [12]. Therefore, it is crucial to introduce effective ways of improving plant growth and reducing such damage. The application of various chemical fertilizers should affect the resistance as well as the tolerance of rice plants to biotic stresses such as the brown plant hopper (BPH) [13]. It has also been shown that the risk of BPH outbreaks in hybrid rice could be increased through the application of high levels of nitrogen fertilizer [8, 14].

Silicon (Si) is one of the most prevalent elements in the soil. Si is beneficial for plant growth and contributes to plant defense mechanisms against multiple biotic and abiotic stresses, including tolerance to salt, drought, and toxic metals [15]. Silicon enhancement stimulates plants to produce phytoalexins and phenolics, consequently increasing the plants' capacity to defend against stress factors [16]. Si may reduce oxidative damage in plants encountering environmental stresses [17], and it plays an important role in enhancing plants' tolerance to biotic stresses, such as fungal diseases. This increased tolerance is partly attributed to increases in the defense capacity of plants due to Si-induced increases in antioxidants. A Si-cuticle double layer is produced due to Si deposition beneath the cuticle and acts as a barrier to physically prevent the penetration of fungi [15]. Si can improve soil conditions in the presence of toxic heavy metals; minimize the toxicity of Mn, Al, and Fe; and enhance P availability [15, 18]. The Si concentration depends on the plant genotype, as it does in other organisms. Therefore, plants' metabolic activities and physiological mechanisms might be affected by different amounts of Si used in fertilizers [15, 19, 20].

Rice, which can accumulate Si up to 10.0% of the shoot dry weight, shows the most effective Si accumulation among plants. Stable production and healthy growth of rice are highly affected by the Si content of the shoots. The high Si accumulation in rice shoots is attributed to the ability of rice roots to take up large amounts of Si [21]. The addition of Si to a susceptible rice variety infected by* Magnaporthe grisea* was associated with the accumulation of momilactone phytoalexins [22] and the upregulation of peroxidase,* PR-1* transcripts, and glucanase [23].

Silicon can increase the tolerance of plants to UV-B stress and increase the amounts of chlorophyll a and b [24, 25]. The amount of Si on the adaxial surface of rice-leaves increases tolerance to UV-B radiation, suggesting that silicic accumulation may help reduce the physical stress caused by UV radiation [26]. It has been reported that Si supplementation during the hydroponic culture of rice enhances UV-B resistance, which might be attributed to increases in phenolic compounds in the plant epidermis [27].

Silicic acid is the form of Si that is absorbable by plant roots; it translocates to the shoots through the transpiration stream and is later polymerized and accumulated as silica in the shoot tissues [28, 29]. Si uptake in rice is controlled by at least two transporters,* Lsi1* (influx) and* Lsi2* (efflux), but the accumulation of Si is mediated only by* Lsi1* [30]. Therefore, it is hypothesized that the transformation of* Lsi1* and its expression or overexpression in Indica rice varieties may improve the resistance of the plant to stress factors. In this study, we screened the* Lsi1* gene in fourteen Malaysian rice varieties. Three common Malaysian rice varieties were selected for genetic manipulation and showed morphological improvements due to greater Si accumulation.

## 2. Experimental Procedures

### 2.1. Plant Material

Fourteen important Malaysian rice varieties (MR219, MR276, MR220, MR211, MR263, MR269, MR185, MR159, MRQ74, MRQ50, Mashuri, Jaya, Masria, and Milyang) were provided by the Malaysian Agricultural Research and Development Institute (MARDI).

### 2.2. Screening of the* Lsi1* Gene

Rice seeds were soaked in water for three days at 25°C and germinated in Petri dishes using Whatman filter paper (Sigma-Aldrich, USA) moistened with a 0.5 mM CaCl_2_ solution. Subsequently, seeds with a bud length of 1-2 mm were selected, transferred into a hydroponic culture solution [31], and kept in a glasshouse at 25–30°C under fluorescent white light with a photoperiod of 16 hours light/8 hours dark for three weeks. Throughout the experiment, the pH of the solution was maintained at 5.7. The nutrient solution was renewed every 3 days. The root tissues of all varieties were sampled, immediately frozen in liquid nitrogen, and then kept at −80°C for total RNA isolation.

### 2.3. RNA Extraction and Semiquantitative RT-PCR

Total RNA from the roots of the fourteen varieties was isolated separately using TRIzol Reagent (Invitrogen, USA). For all samples, DNase I (Qiagen, USA) was used to digest contaminating genomic DNA. First-strand cDNA was synthesized from each sample using SuperScript III (Invitrogen, USA). The Taq DNA polymerase kit (Fermentas, Waltham, Massachusetts, USA) was used with the following program: initial denaturation at 95°C for 5 min, 35 cycles of 95°C for 30 sec, annealing (55°C, 56°C, 57°C, 58°C, 59°C, 60°C, 61°C, 62°C, 63°C, 64°C, 65°C, and 66°C) for 45 sec, extension at 72°C for 1 min, and a final extension for 10 min at 72°C. The PCR products were isolated on 1.5% agarose gels and stained with Midori Green (Nippon Genetics, Germany). Two sets of specific primers were designed for the* Lsi1* gene based on the sequence available in GenBank using Primer Premier 6 (Table 1).

### 2.4. Construction of the Expression Clone

#### 2.4.1. Preparation of the attB PCR Product

**Figure 1:**
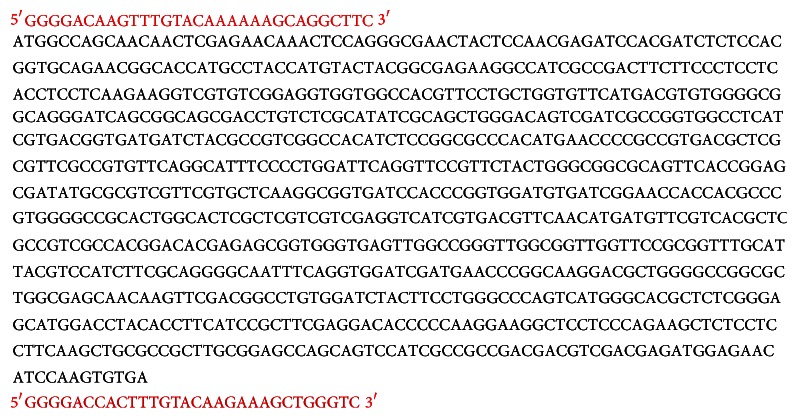
The CDS of* Lsi1* with one pair of anchors (required to make the attB PCR product) at the 5′ and 3′ ends (nucleotides shown in red).

The CDS of the* Lsi1* gene includes 897 bp (GenBank: AB222272.1). One pair of adaptors (anchors) was designed according to the instructions provided by the manufacturer of the Gateway® Technology with Clonase™ II (Invitrogen, USA) kit based on the structure of the pDONOR/Zeo vector. The kit and vector were provided by First BASE Laboratories Sdn Bhd, Malaysia (Figure 1).

The template sequence was amplified using the following specific primers:

Forward primer: 5′ GGGGACAAGTTTGTACAAAAAAGCAGG 3′
Reverse primer: 5′ GACCCAGCTTTCTTGTACAAAGTGG 3′

The following PCR (KAPA HiFi Hot Start DNA polymerase kit, USA) program was performed: 95°C for 3 min and 30 cycles of 95°C for 30 sec, 62°C for 30 sec, and 72°C for 30 sec, followed by a final extension of 10 min at 72°C. The PCR product was isolated on a 1.5% agarose gel and stained with Midori Green (Nippon Genetics, Germany). The expected 958-bp band was purified using a QIAprep® Spin miniprep kit (Qiagen, Germany) and submitted for sequencing.

The Gateway Technology kit (Invitrogen, USA) was used to run the BP and LR recombination reactions according to the manufacturer's procedures using the expression vector pFast G02 [32].

#### 2.4.2. Transformation of Competent Cells

Transformation was performed using the* E. coli* strain TOP10, which lacks the ccdA gene and is not sensitive to the effects of the ccdB gene. The ccdB gene, which is found in the structure of the pDONOR™/Zeo vector, functions as a negative selectable marker. Zeocin was used for bacterial selection in low-salt LB agar. The transformation process was performed according to the instructions provided by the manufacturer of the PCR Cloning^plus^ Kit (Qiagen, Germany).

#### 2.4.3. *Agrobacterium*-Mediated Transformation

The* Agrobacterium tumefaciens* strain LBA4404 was used to transfer the expression clone containing the CDS of the* Lsi1* gene to the calli of three selected varieties, MR219, MR220, and MR276, via the freeze-thaw method.

### 2.5. In Vitro Studies, Part One

The mature dry seeds of elite Indica rice varieties (MR219, MR220, and MR276) selected from the screening were used. The hulled seeds were sterilized using 70% alcohol and shaken at 120 rpm for 20 min in a 40% solution of sodium hypochlorite containing three drops of polyoxyethylene sorbitan monooleate using an orbital shaker. The seeds were rinsed five times with autoclaved distilled water. Finally, the seeds were dried on autoclaved filter paper and transferred to the culture room. Ten sterile seeds of each variety were placed separately in two Petri dishes (100 × 15 mm) containing MS-2,4-D medium, pH 5.8. The cultured seeds were then incubated at 25°C in the dark for three weeks until a yellowish white embryogenic callus appeared on the scutellar surface.

#### 2.5.1. Transformation of MR219, MR220, and MR276 Embryogenic Calli with* Agrobacterium* Containing the pFast G02 Vector

The OD of an* Agrobacterium* suspension culture was adjusted to 0.8 using N6-AS medium. Then, embryogenic calli from three varieties were placed in the* Agrobacterium* suspension for 30 min in three different Petri dishes. Subsequently, bacterial suspension from each Petri dish was pipetted out, and the excess* Agrobacterium* infection medium was absorbed with autoclaved filter paper. The transformed calli of all three varieties were then transferred to N6-AS medium (solid cocultivation medium). Finally, the three plates were incubated at 28°C for 72 hours in the dark.

Transgenic plants were generated using the following steps [3]: production of embryogenic calli in MS-2,4-D at 25°C for 3 weeks under dark conditions; proliferation of transformed calli in MS-2,4-D + cefotaxime at 25°C for 10 days under dark conditions; selection of transformed calli using MS-2,4-D + cefotaxime + BASTA (first selection) at 25°C for 2 weeks; selection of transformed calli using MS-2,4-D + cefotaxime + BASTA (second selection) at 25°C for 2 weeks; selection of transformed calli using MS-2,4-D + cefotaxime + BASTA (third selection) at 25°C for 2 weeks; shoot production from surviving and healthy embryogenic calli in MSKN regeneration medium at 25°C for 20 days under dark conditions; plantlet regeneration in regeneration medium (MSKN) at 27°C under light conditions (with a 16-hour photoperiod) for 20 days; root production in rooting medium (MSO) at 27°C under light conditions (with a 16-hour photoperiod) for 20 days.

#### 2.5.2. Transfer of Transgenic MR219, MR220, and MR276 Plants to the Soil

The culture medium from the roots of the plantlets was gently removed after two weeks, when the root system was well developed. Then, the plantlets were placed in Yoshida culture solution and transferred to a transgenic greenhouse with 95% relative humidity and a 14-hour photoperiod (160 *μ*mol/m^2^/s) and grown at 29°C for three weeks. Then, plants with dynamic root systems were put into pots containing paddy soil, water (initially 1 L per pot), and fertilizers. All pots received a basal application of 50 kg N ha^−1^, 70 kg P ha^−1^, and 70 kg K ha^−1^. The sources for N, P, and K were urea (60% N), KH_2_PO_4_ (28.6% K and 22.7% P), and muriate of potash (MoP) (50% K), respectively. N fertilizer top dressing (50 kg N ha^−1^ N) was performed at the tillering and flowering stages. When the color of the grains (almost 85%) turned straw gold, panicles from each variety were harvested.

#### 2.5.3. Analysis of Transgenic MR219, MR220, and MR276 Plants

The seeds of transformed plants kept in the transgenic greenhouse were harvested after full maturation. The putative transgenic plants were analyzed using the 3G plant PCR kit, KAPA (South Africa), with the following program: initial denaturation at 95°C for 3 min, 35 cycles of 95°C for 30 sec, annealing (54°C for* Lsi1* and 60°C for* CaMV35S*) for 45 sec, extension at 72°C for 1 min, and a final extension at 72°C for 10 min. The PCR amplification was performed using two sets of specific primers (Table 2).

#### 2.5.4. *GFP* Monitoring of MR219, MR220, and MR276 Transgenic Seeds

*GFP* expression was detected in the transgenic rice seeds obtained from the T_0_ and T_1_ generations using a fluorescence microscope (Leica MZFL111) adjusted with a GFP2 filter. Images of transgenic seeds were captured using the fluorescence microscope with the Leica DC 200 system and its related software (Leica DC Viewer).

### 2.6. In Vitro Studies, Part Two

Mature dry seeds of transgenic rice varieties (MR219, MR220, and MR276) were used. The procedure described in part one of the in vitro study was followed. Three sterile seeds of each variety were placed separately in three glass vials containing MS media supplemented with 2 mg L^−1^ 2,4-D and 3.5 mM K_2_SiO_3_, pH 5.8. Three replicates of each glass were measured; thus, a total of 9 glass vials were examined. The cultured seeds were then incubated at 25°C in the dark for three weeks until a yellowish white embryogenic callus appeared on the scutellar surface. When the regeneration steps of the shoots and roots of the transgenic plants were completed following the steps described in part one of the in vitro study, the transgenic plants were transferred to the transgenic greenhouse.

#### 2.6.1. Expression Analysis of the* Lsi1* Gene in T_1_ Transgenic Plants Using Real-Time PCR

Total RNA was extracted from both wild-type and transformed plants using TRIzol. Real-time qRT-PCR (KAPA SYBR FAST One-Step qRT-PCR, USA) was performed to assess the expression level of the candidate gene* (Lsi1)* in wild-type and transgenic plants (MR219, MR220, and MR276). The following program was used for real-time qRT-PCR: 42°C for 5 min, inactivation of RT at 95°C for 5 min, followed by 40 cycles at 95°C for 3 sec, 60°C for 30 sec, and 72°C for 3 sec. The data were obtained from three independent biological replicates. Amplicon specificity was controlled using a melting curve analysis through increasing the temperature from 60°C to 95°C after 40 cycles.* Lsi1* and two internal reference genes (*18S rRNA* and *α-Tubulin*) were amplified using specific primers (Table 3). The REST software (Qiagen, Hilden, Germany) was hired to analyze the results of the qRT-PCR and all data were normalized using ΔΔCT method. The 18S rRNA and *α-Tubulin* reference genes of* O. sativa* were used as expression reference genes for normalization. The manufacturer's instruction was followed to quantify the relative gene expression.

#### 2.6.2. Estimation of Antioxidant Enzyme Activity

Regenerated root and leaf tissues (100 mg) from each transgenic variety were homogenized separately in phosphate buffer (50 mM, pH 7.0) containing polyvinylpyrrolidone (1.0 mM), Triton X-100 (0.05%), EDTA (1.0 mM), and ascorbate (1.0 mM). Then, the homogenate was centrifuged at 13500 rpm at 4°C for 15 min. Next, the activities of antioxidant enzymes were measured using the supernatant. The activity levels of superoxide dismutase (SOD), peroxidase (POD), ascorbate peroxidase (APX), and catalase (CAT) were determined using previously published methods [33].

#### 2.6.3. Chlorophyll Content

The leaves of wild-type and transgenic plants were collected separately for each cultivar. The chlorophyll contents (a, b, and total) were estimated using the method of Arnon [34]. Approximately 0.5 g of plantlet leaves was homogenized in liquid nitrogen and then solubilized in 80% acetone. Then, the mixture was centrifuged at 10,000 rpm for 15 min. Finally, the supernatant was collected, and the absorbance at 663 and 645 nm was measured using a scanning spectrophotometer (AL800, Aqualytic, Germany). The chlorophyll contents were measured as mg/g of sample.

#### 2.6.4. Analysis of Photosynthesis

The LI-6400XT portable photosynthesis system (LI-COR, Lincoln, Nebraska, USA) was used to measure and compare the photosynthesis of wild-type and transgenic varieties after they were transferred to the soil.

#### 2.6.5. Silicon Concentration

Determination of the amount of Si in each of the three transgenic varieties compared to the wild-type plants was conducted following the method described by Korndörfer et al. [35]. The leaves and roots of three transgenic varieties (T_1_) were dried at 60°C for 72 hours using a ventilated oven. When the samples reached a constant weight, they were ground separately. For the digestion step, 0.1 mg of dried and ground sample was placed in a polyethylene tube, followed by the addition of 2.0 mL H_2_O_2_ and 3.0 mL of NaOH (25 mol L^−1^) and incubation at 123°C and 0.15 MPa for 1 hour using an autoclave. After the tubes were cooled, deionized water was added to fill each tube. Finally, an aliquot (1.0 mL) of this extract was taken, and 20 mL of deionized water was added to monitor the Si concentration using a spectrophotometric method. Silicon reacts with the ammonium molybdate in the HCl medium, resulting in the formation of molybdosilicic acid and the emission of a yellow color that can be detected by spectrophotometry at 410 nm.

#### 2.6.6. Si Observations under Scanning Electron Microscopy

This part of the experiment was performed using the calli and roots of both wild-type and transgenic rice varieties. Calli of wild-type and transgenic varieties cultured in 1 *μ*M-silicate medium (MS medium + K_2_SiO_3_) for 45 days were dehydrated by filtration through an ethanol series and then freeze-dried (JFD-300, JEOL, Tokyo, Japan). Similarly, the roots of wild-type and transgenic rice varieties obtained from hydroponic cultures supplemented with 1.5 mM SiO_2_ were dehydrated and dried using a critical point dryer (CPD2, Pelco, CA, USA). Then, the dried samples were coated with gold on aluminum stubs using a sputter coater (SC7640, Polaron, Sussex, UK). Samples were observed under scanning electron microscopy (SEM) (LEO 1455 VPSEM, New England). Images were obtained at 20.00 kV and at magnifications of 350, 400, and 500x.

#### 2.6.7. Morphological Characteristics of Transgenic Varieties

In this part of the experiment, we used transgenic varieties and wild-type rice varieties in dry seeding and wet seeding. Seven basic characteristics, root length, root dry weight at the maximum tillering stage, root dry weight at the panicle initiation stage, root dry weight at the flowering stage, shoot dry weight at the maximum tillering stage, shoot dry weight at the panicle initiation stage, and shoot dry weight at the flowering stage, were measured for comparisons of transgenic and control plants.

### 2.7. Statistical Analysis

The experiments were repeated three times for each transgenic variety and wild-type parent. The normalized data were statistically analyzed (ANOVA and LSD multiple test) using SAS software (version 9.1, USA).

## 3. Results of Screening and Expression of the* Lsi1* Transgene

### 3.1. *Lsi1* Screening

The screening of the* Lsi1* between Malaysian rice cultivars showed that this gene is not existed in the 14 wild-type varieties using two sets of specific primers with a series of annealing temperatures.

### 3.2. Analysis of T_1_ Transgenic MR219, MR220, and MR276 Rice Varieties

**Figure 2:**
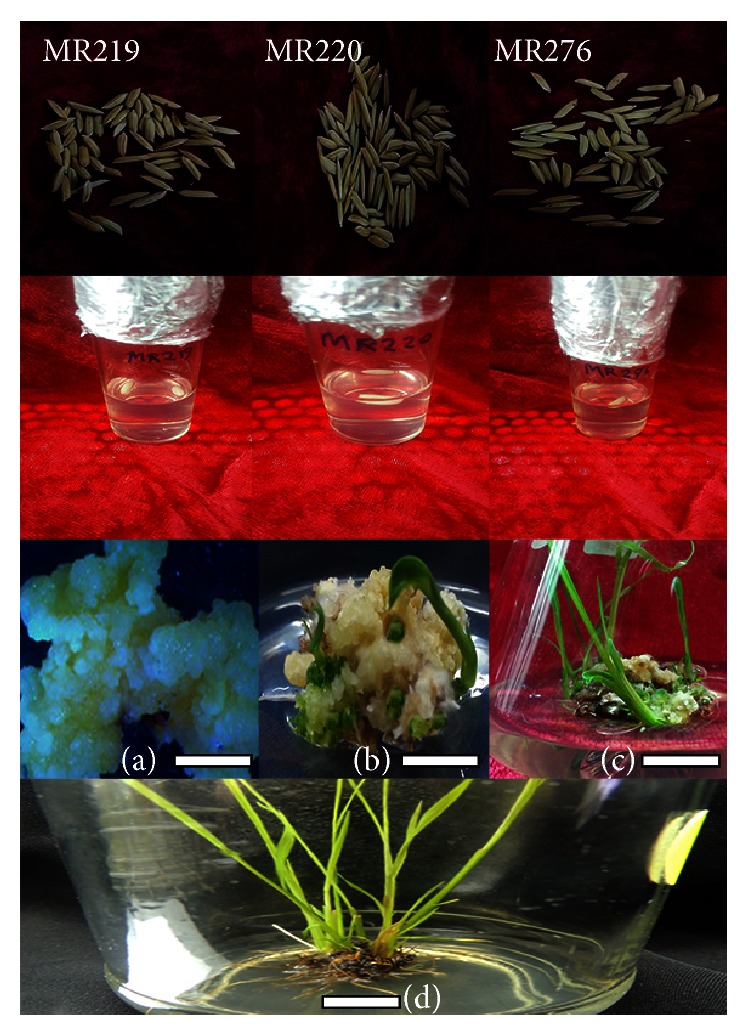
Regeneration of T_1_ seeds of transgenic MR219, MR220, and MR276 varieties. (a) Callus induction; (b) and (c) regenerated transgenic shoots; (d) regenerated transgenic roots. Bars = 1 cm.

Three and ten plants of MR219, five and nine plants of MR220, and four and eight plants of MR276 were identified as the T_0_ and T_1_ transgenic generations, respectively. Three transgenic varieties, MR219, MR220, and MR276, were submitted to NCBI (KR673322, KT284742, and KT284741). The regenerated plant seeds (T_1_) continued to grow through in vitro culture and after planting in the soil in the transgenic greenhouse (refer to Figure 2 in the methodology, part two of the in vitro study).

**Figure 3:**
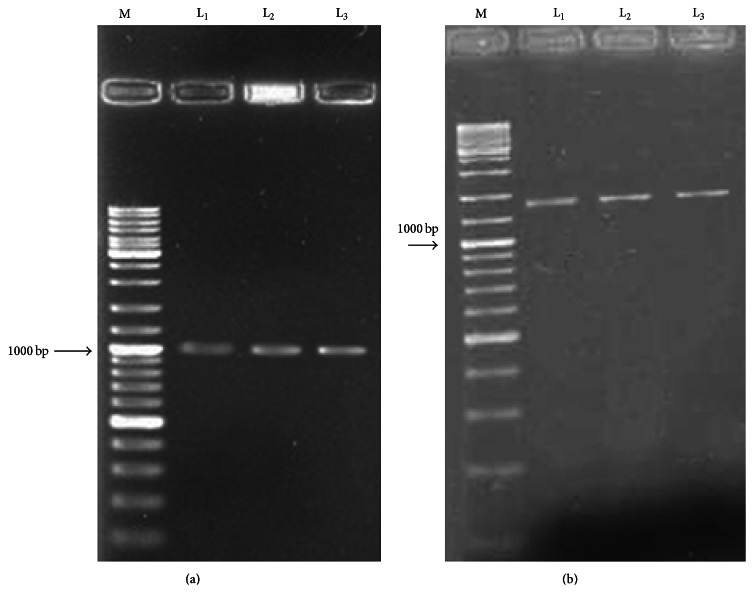
Detection of T_1_ putative transgenic plants of MR219, MR220, and MR276 ((a) using specific primers, (b) using* CaMV35S* primers). M: 1 kb DNA ladder; lanes 2-3: the* Lsi1* gene in transgenic MR219, MR220, and MR276 plants.

The results obtained from the analysis of each T_1_ transgenic MR219, MR220, and MR276 plant gave two specific bands of 897 and 1198 bp (Figure 3).

### 3.3. *GFP* Monitoring of the T_0_ Transgenic Calli and T_1_ Transgenic Seeds of MR219, MR220, and MR276

**Figure 4:**
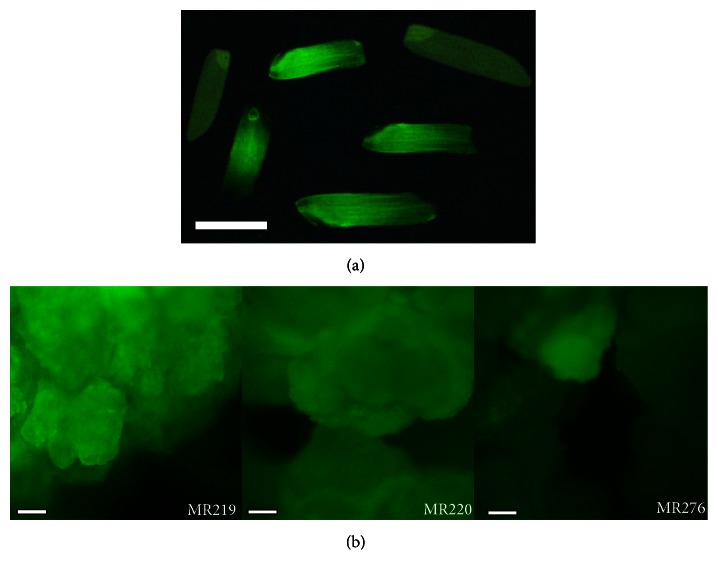
Expression of* gfp* among harvested seeds (a) and calli (b) obtained from T_1_ transgenic MR219, MR220, and MR276 varieties. The green color observed under fluorescence microscopy is related to expression of green fluorescent protein* (GFP)*, confirming the presence of the transgene. Bars = 5 mm.

The seeds harvested from plants of the T_0_ and T_1_ generations (Figure 4(a)) and calli obtained from the T_1_ transgenic plants (Figure 4(b)) were monitored under a fluorescence microscope to record* gfp* expression. The seeds of the T_0_ generation expressing* gfp* were selected and used to obtain the T_1_ generation, followed by the selection of T_1_ seeds expressing* gfp* for further analysis. Meanwhile, some of the calli obtained from T_1_ transgenic plants expressing* gfp* were transferred to culture in a medium supplemented with K_2_SiO_3_.

### 3.4. Differential Expression of the* Lsi1* Gene in Transgenic Plants

**Figure 5:**
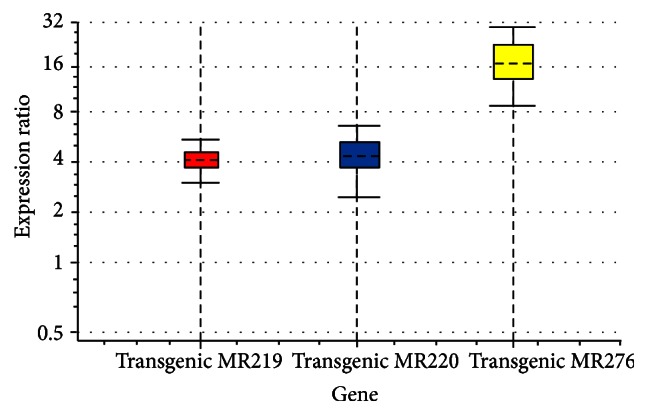
Relative expression levels of the* Lsi1* gene were calibrated using quantitative real-time PCR of two reference genes,* 18S rRNA* and *α-Tubulin*, in wild-type and transgenic varieties. Expression of* Lsi1* in the transgenic cultivar MR276 was significantly higher than in the transgenic cultivars MR219 and MR220.

The real-time qRT-PCR analysis indicated that the expression level of the* Lsi1* gene was higher in all transgenic varieties compared to their related wild-type or untransformed plants. To avoid positional effects of transgenesis, the expression level of* Lsi1* was considered using a constitutive promoter* (CaMV35S)* and the same tissue (roots) in all transgenic varieties. Differences among transgenic varieties were statistically significant (*P* < 0.01). The standard curves showed high correlation coefficient of *R*^2^ = 0.994, 0.995, and 0.997, presenting that the Ct values were proportional to the copy numbers of transgene for all three varieties. The amplification efficiencies of transgene in three varieties were 97.3, 98.4, and 98.7%. The standard curve of transgene for each variety was within the acceptable range (amplification efficiency of 90–110% and *R*^2^ value of 0.985). The upregulation of the* Lsi1* gene in transgenic MR276 was higher than that in the transgenic MR219 and MR220 plants (Figure 5).

### 3.5. Si Concentration and Observation under Scanning Electron Microscopy

**Figure 6:**
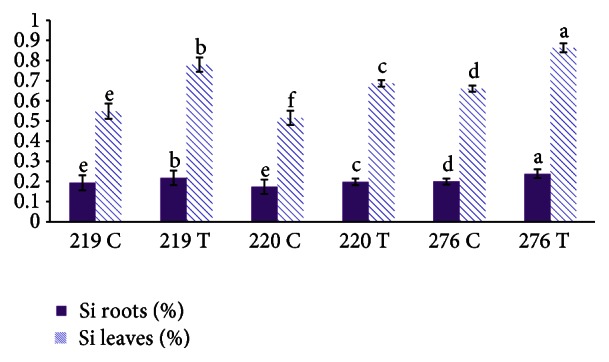
Comparison of Si concentrations in transgenic and wild-type rice varieties. T: transgenic. C: control.

Si concentrations in the roots and leaves were significantly higher in transgenic plants than in wild-type plants (Table 4). The Si concentrations in the roots and leaves of transgenic MR276 were higher than those in the transgenic MR219 and MR220 varieties. The MR220 variety, among all the transgenic plants, showed the smallest changes in Si concentration extracted from the leaves and roots. These results strongly confirmed the results obtained from real-time qRT-PCR (Figure 6), suggesting that the transgenic MR276 variety, with a higher expression level of the* Lsi1* gene, absorbed more Si from the culture medium as well as from the hydroponic culture. The bar graph shows that all of the wild-type varieties absorbed some amount of Si from the culture medium or hydroponic culture, confirming the ability of rice plants to absorb Si. Moreover, these results demonstrate that the wild-type forms of these three varieties also differed from each other with respect to Si absorption. It can be concluded that regardless of the presence of the transgene, the MR276 plants absorbed more Si than did the corresponding MR219 and MR220 plants.

**Figure 7:**
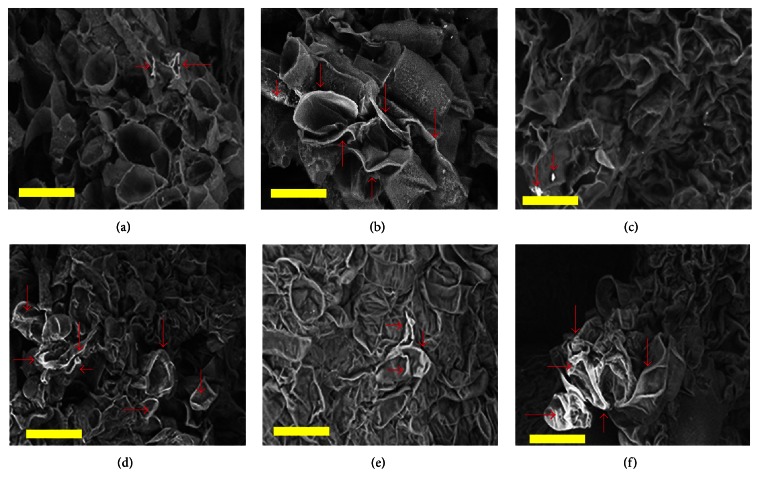
Scanning electron microscopy images of wild-type and transgenic calli. (a) Callus of wild-type MR219. (b) Callus of transgenic MR219. (c) Callus of wild-type MR220. (d) Callus of transgenic MR220. (e) Callus of wild-type MR276. (f) Callus of transgenic MR276. White trace marked by red arrows: silicon. Bars = 100 *μ*m.

**Figure 8:**
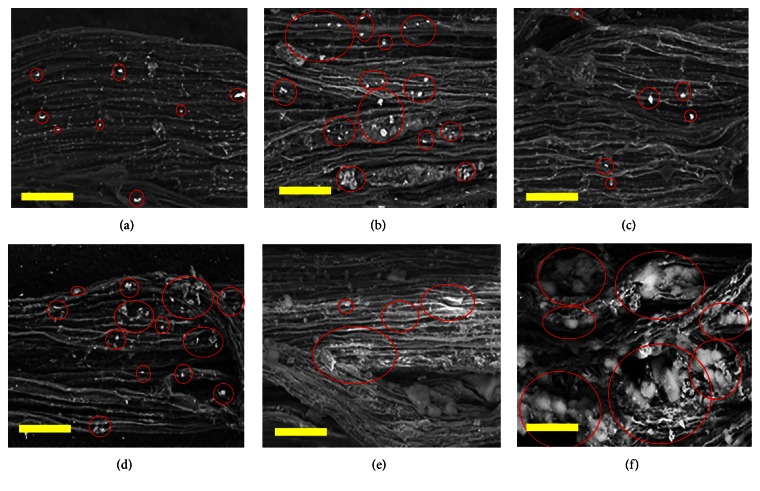
Scanning electron microscopy images of wild-type and transgenic rice roots. (a) Roots of wild-type MR219. (b) Roots of transgenic MR219. (c) Roots of wild-type MR220. (d) Roots of transgenic MR220. (e) Roots of wild-type MR276. (f) Roots of transgenic MR276. White spot marked by red circle: silicon. Bars = 100 *μ*m.

Electron microscopy images of the roots and calli of the transgenic varieties showed that the Si concentration was higher in all transgenic varieties compared to wild-type varieties, although the level differed between transgenic varieties (Figures 7 and 8). Roots and calli obtained from T_1_ transgenic MR276 absorbed more Si than did those of the transgenic MR220 and MR219 varieties. The images in Figure 8 strongly confirm the results obtained from examining the Si concentration in the roots of plants, shown in Figure 6. Electron microscopy images show that the MR276 variety (both wild-type and transgenic forms) has a higher capacity for Si absorption from the media or hydroponic culture than the other two varieties, with MR220 showing the next-highest level of Si absorption.

### 3.6. Antioxidant Enzyme Activities, Chlorophyll Content, and Photosynthesis Analysis

**Figure 9:**
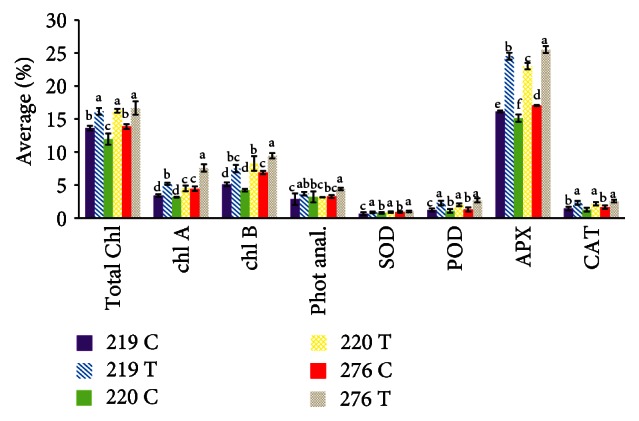
Variations in antioxidant activities and physiological traits among transgenic and wild-type rice varieties. The same small letter(s) in different columns denote no significant difference at *P* ≤ 0.05. Total Chl.: total chlorophyll content. Chl. A: chlorophyll A. Chl. B: chlorophyll B. Phot anal.: photosynthesis analysis. SOD: superoxide dismutase. POD: peroxidase. APX: ascorbate peroxidase. CAT: catalase. T: transgenic. C: wild-type (control).

The transgenic varieties showed significant increases in the activities of the enzymes SOD, POD, APX, and CAT; the chlorophyll content; and photosynthesis compared with the wild-type plants (Figure 9, Table 4). Superoxide dismutase, catalase, and peroxidase activities in all three transgenic varieties showed the same changes. However, the transgenic MR276 had the highest ascorbate peroxidase activity of the transgenic varieties.

The total chlorophyll content in the wild-type MR276 was the same as that in the wild-type MR220, and the total chlorophyll content in the wild-type MR219 was higher than that in the wild-type MR220. However, all three transgenic varieties showed the same increase in total chlorophyll content. Chlorophyll A and B levels were higher in transgenic MR276 than in transgenic MR219 and MR220. Chlorophyll A in transgenic MR219 was higher than that in transgenic MR220; however, in both transgenic varieties, chlorophyll B showed the same changes compared to wild-type plants. The photosynthetic changes in both transgenic MR276 and transgenic MR219 were the same and represented greater changes in photosynthesis than in transgenic MR220.

### 3.7. Morphological Characteristics

**Figure 10:**
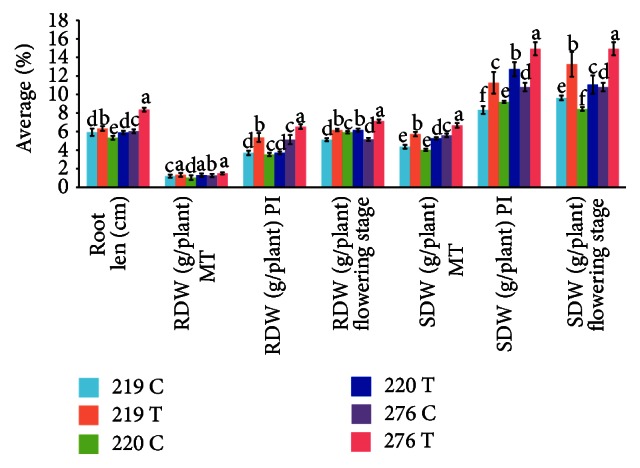
Functional effects of the transgene on various characteristics of rice.

**Figure 11:**
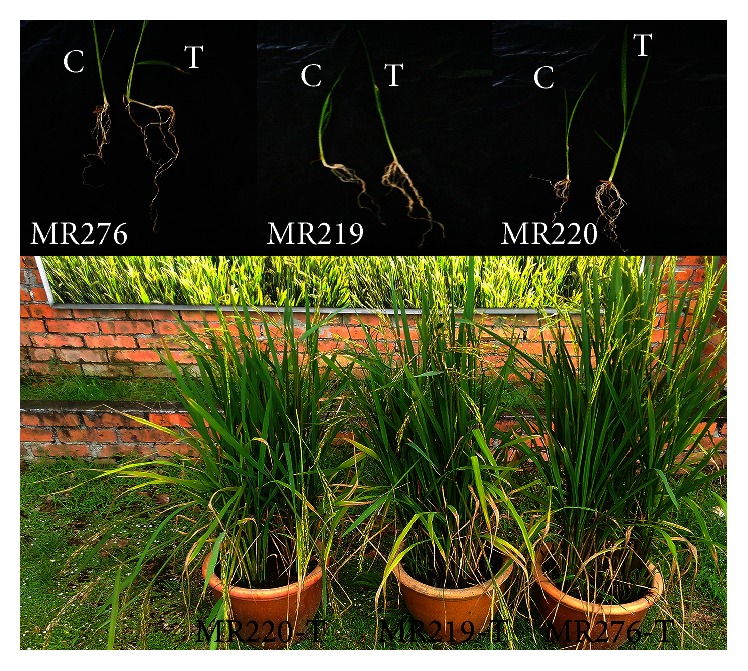
Morphological comparison between wild-type (control) and transgenic plants. T: transgenic. C: control.

The data presented in Figure 10 and Table 4 demonstrate that the root length, root dry weight at the maximum tillering stage (RDW (g/plant) MT), root dry weight at the panicle initiation stage (RDW (g/plant) PI), shoot dry weight at the maximum tillering stage (SDW (g/plant) MT), shoot dry weight at the panicle initiation stage (SDW (g/plant) PI), and shoot dry weight at the flowering stage (SDW (g/plant)) were significantly different between transgenic and wild-type varieties. The root dry weight at the panicle initiation stage (RDW (g/plant) PI) recorded for all transgenic varieties was similar, whereas the transgenic MR276 variety had a markedly higher value for the other six morphological traits. The values of root length, root dry weight at the panicle initiation stage (RDW (g/plant) PI), root dry weight at the flowering stage (RDW (g/plant)), shoot dry weight at the maximum tillering stage (SDW (g/plant) MT), and shoot dry weight at the flowering stage (SDW (g/plant)) for transgenic MR219 were higher than those for transgenic MR220. However, the shoot dry weight at the panicle initiation stage (SDW (g/plant) PI) recorded for transgenic MR220 was higher than that for transgenic MR219. The root dry weight at the flowering stage did not differ between the transgenic MR219 and MR220 varieties. Overall, the lowest values were recorded for the transgenic MR220 variety, and the highest values were recorded for the transgenic MR276 variety. Transgenic varieties had a stronger root and leaf structure, as well as hairier roots, compared to the wild-type plants (Figure 11). The transgenic MR220 variety had hairier but shorter roots compared to the transgenic MR219 and MR276 varieties.

## 4. Discussion

This is the first study to illustrate the effect of* Lsi1* expression on morphological and physiological properties in Indica rice varieties. Our screening assessment revealed that the* Lsi1* gene is not present in fourteen wild-type Malaysian rice varieties. However, previous studies have identified the* Lsi1* gene in the mutant Japonica rice variety and one Indica rice cultivar (Dular) [21, 36]. The* Lsi1* gene has been identified as a Si transporter in rice, although we could not detect this gene among these fourteen varieties in wild-type form. However, our evaluation confirmed the accumulation of Si among the wild-type Malaysian rice varieties, which may be due to the expression of an unknown gene family. Alternatively, it can be hypothesized that the* Lsi1* gene is only expressed in rice under specific treatments, such as sodium azide [21], which explains why we did not observe this gene in wild-type rice varieties. In that case we have evaluated the overexpression of the* Lsi1* gene in the Indica rice variety. It should be noted that* Lsi1* plays an important role in Si accumulation in transgenic varieties of rice, although the effective expression level of this gene was different between all transgenic rice varieties. Moreover, the effects of this gene were not entirely limited to increasing Si in the roots and leaves of transgenic varieties. In vitro studies of the roles of Si in plant tissue culture concluded that Si can significantly increase root length, chlorophyll content, fresh and dry weights of roots and shoots, and plant height [37–39]. The ability of Si to enhance plant growth and development is based on alterations in antioxidant enzyme activities [37]. In this regard, it has been reported that Si treatment considerably affects antioxidant enzyme activities in various plants [40, 41]. Although most plant scientists believe that Si is not essential for plant growth and development and do not consider Si to be a plant nutrient, the results obtained from this study strongly emphasize the crucial role of Si in plant growth and development.

In our study, the expression of the* Lsi1* gene in transgenic rice varieties helped these plants to absorb more Si compared to the wild-type plants, as confirmed by morphological analysis. The transgenic plants with more accumulated Si had higher antioxidant enzyme activities and improved morphological traits. The morphological analysis of transgenic rice varieties provided results that strongly confirm the results of previous studies. We hypothesized that the low Si uptake in wild-type Malaysian rice varieties may be due to either the absence or the low expression of the* Lsi1* gene. Our screening observations and the studies of transgenic varieties confirmed this hypothesis.

Genetic modification is a new technique that has been used by researchers to increase plant yields via trait improvement [42]. Instead of using Si as a fertilizer or as a culture medium supplement in in vitro studies, we tried to genetically manipulate plants to improve their Si uptake. Such transgenic plants should be able to absorb more Si through an improved root system. We observed a positive correlation between Si accumulation and photosynthesis components, as well as consequent improvements in morphological characteristics, in transgenic rice plants.

Indeed, increases in chlorophyll content and photosynthesis are typical responses of plants supplemented with Si [24, 43]. There are many explanations as to how Si accumulation leads to alterations in antioxidant enzyme activities, increases in chlorophyll content and photosynthesis, and improvements in morphological properties. In one study, higher levels of Si in rice suppressed drought stress by improving the plant's water status, mineral nutrient uptake, and photosynthesis [44]. Silicon enhanced resistance to freezing stress in wheat, which is likely attributable to the protective effects of higher antioxidant levels and lower lipid peroxidation via water preservation in leaf tissues [45]. An assessment of the effect of Si on antioxidant enzymes in cotton showed that Si altered the activities of guaiacol peroxidase (GPOX), catalase (CAT), and ascorbate peroxidase (APX) in roots and leaves, whereas lipid peroxidase activity was not affected. These authors reported that enzymatic activities in the roots and leaves of plants were changed in parallel with silicon supplementation [46].

Quantitative real-time PCR is a versatile technique for sensitive, reliable, precise, and high-throughput detection and quantification of a target sequence in different samples [47]. Although the expression of* Lsi1* in transgenic plants leads to improved growth and development of transgenic varieties based on the results of real-time qRT-PCR, it can be concluded that this gene is differentially expressed in different transgenic varieties. Therefore, the positive results obtained from the expression of the* Lsi1* gene among three Indica rice varieties cannot be predicted or expected in other plant species, and this phenomenon needs to be examined for individual plant species. In this study, we performed qRT-PCR of the Si transporter response gene* (Lsi1)* to evaluate its expression levels in the transgenic forms of three commonly grown local rice varieties. Initially, it seemed that all transgenic varieties showed the same effects, whereas the results of qRT-PCR showed that the transgenic MR276 rice variety had highest* Lsi1* expression level. We could not find any reasons for these differences between the transgenic varieties. We believe that the results obtained from further experiments, which demonstrated that transgenic MR276 showed high levels of antioxidant enzyme activity and improved morphological properties, are not directly due to the expression of* Lsi1*. The transgenic MR276 variety absorbed more Si than the other transgenic varieties, so it is likely that other properties of this transgenic variety are related to increased accumulation of Si in the roots and leaves of the plant. However, the expression of only one target gene cannot be effective in creating diverse traits. It must be mentioned that the activation of the* Lsi1* gene at the same time promoted Si accumulation and may have affected antioxidant enzyme activities by producing plant tonoplast intrinsic proteins (TIPs). Various members of the TIP family, including alpha (seed), Rt (root), gamma, and Wsi (water stress induced), may be effective in altering antioxidant enzyme activities. It has also been predicted that these protein family members may allow the diffusion of different amino acids, peptides, and water from the tonoplast center to the cytoplasm. On the other hand, stimulating the diffusion of some amino acids, such as serine and proline, should be more effective for biosilica formation by plants [48].

Our results suggest that the transformation of the* Lsi1* gene into Indica rice varieties (overexpression or as a foreign gene) is more effective than supplementing the culture medium or soil with Si. Changes in antioxidant enzyme activities, chlorophyll content, photosynthesis, and morphological properties were significantly beneficial in transgenic varieties, while the wild-type varieties were also treated with the same source and amount of Si in the culture medium and the hydroponic culture solution. Although the transgenic varieties did not all show the same variation, the transgenic MR220 variety, which showed the smallest changes, still showed better characteristics than any of the wild-type varieties.

Finally, based on the function of the* Lsi1* protein in the mechanism of diffusion of various amino acids from the tonoplast interior to the cytoplasm and on the results of our previous study, which confirmed the role of* serine-rich protein* in Si accumulation by* Arabidopsis thaliana* as a model plant [48], a future study comparing the roles of* Lsi1* and* serine*/*proline-rich protein* in Si uptake by plants is needed.

## 5. Conclusion

Silicon plays an important role in plant growth and development. Although Si is plentiful in the soil and does not need to be applied as fertilizer, different plant species differ in Si uptake from the soil. The* Lsi1* gene has been identified as a Si transporter gene in the Japonica rice variety. Our screening did not detect the* Lsi1* gene among Indica rice varieties. Based on the important role of this gene in Si uptake by plants and the crucial role of Si in plant growth and development, we tried to increase Si absorption and accumulation inside the Indica rice varieties via genetic manipulation rather than using additional different sources of Si in the culture medium or soil. The rice variety MR276 showed a more positive response to genetic manipulation than the other varieties, which will aid in future analyses. For instance, because it has been reported that Si can alleviate various stresses such as drought, salinity, and pathogen attack in plants, the transgenic MR276 developed here should be more stable under such harsh environmental conditions. In conclusion, if we can provide genetically manipulated plants that are capable of taking up more Si from the soil, their improved morphological traits and improved resistance against various biotic and abiotic stresses will improve their yields.

## Figures and Tables

**Table 1:** Primers for the *Lsi1* gene used for screening via semiquantitative RT-PCR.

Gene	Forward primer (5′-3′)	Reverse primer (5′-3′)	Expected PCR product size
*Lsi1 *(I)	CCAGGGCGAACTACTCCAACGA	GCTTTGGTTGCTTGGTGGTTCG	1066 bp
*Lsi1 *(II)	ATGGCCAGCAACAACTCG	TCACACTTGGATGTTCTC	897 bp

**Table 2:** Primers used to verify the transgenic MR219, MR220, and MR276 plants.

Gene	Forward primer (5′-3′)	Reverse primer (5′-3′)	PCR product size
*Lsi1*	ATGGCCAGCAACAACTCG	TCACACTTGGATGTTCTC	897 bp
*CaMV35S*	CCGACAGTGGTCCCAAAGAT	TACTCATTTTACTTCTTCG	1198 bp

**Table 3:** Primers used for real-time qRT-PCR of *Lsi1* and two reference genes (*18S rRNA *and *α-Tubulin*).

Gene	Forward 5′ → 3′	Reverse 5′ → 3′
*Lsi1*	ATGGCCAGCAACAACTCG	TCACACTTGGATGTTCTC
*18S rRNA*	ATGATAACTCGACGGATCGC	CTTGGATGTGGTAGCCGTTT
*α-Tubulin*	GGAAATACATGGCTTGCTGCTT	TCTCTTCGTCTTGATGGTTGCA

**Table 4:** Comparisons of various physiological and morphological traits in transgenic and wild-type rice varieties.

SOV	df	Total Chl. Co. (mg/gr)	Chl. A	Chl. B	Photo. Anal.	SOD	POD	APX	CAT	Si% (leaf)
Varieties	5	12.8^*∗∗*^	7.6^*∗∗*^	11.3^*∗∗*^	0.89^*∗∗*^	0.5^*∗∗*^	1.2^*∗∗*^	64.2^*∗∗*^	0.81^*∗∗*^	0.007^*∗∗*^
Replicate	2	0.10^ns^	0.11^ns^	0.05^ns^	0.61^ns^	0.0002^ns^	0.01^ns^	0.19^ns^	0.003^ns^	0.00001^ns^
Error	10	0.37	0.11	0.33	0.18	0.0003	0.006	0.1	0.004	0.00003
Total	17	—	—	—	—	—	—	—	—	—

CV	—	4.1	7.1	8.3	12.42	3.9	4.55	2.1	3.6	15.6

SOV	df	Si% (root)	Root length (cm)	RDW (g/plant) MT	RDW (g/plant) PI	RDW (g/plant) flowering stage	SDW (g/plant) MT	SDW (g/plant) PI	SDW (g/plant) flowering stage	

Varieties	5	0.05^*∗∗*^	3.34^*∗∗*^	0.071^*∗∗*^	4.40^*∗∗*^	1.6^*∗∗*^	2.7^*∗∗*^	17.2^*∗∗*^	17.0^*∗∗*^	
Replicate	2	0.001^ns^	0.15^ns^	0.015^ns^	0.08^ns^	0.001^ns^	0.003^ns^	3.75^ns^	3.49^ns^	
Error	10	0.003	0.13	0.12	0.81	0.007	0.02	1.5	1.3	
Total	17	—	—	—	—	—	—	—	—	

CV	—	9.3	5.8	8.9	6.13	1.47	2.8	11.1	10.3	

ns and  *∗∗* indicate not significant and significance level of 1%, respectively. Total Chl. Co = total chlorophyll content, Chl. A = chlorophyll A, Chl. B = chlorophyll B, Photo. Anal. = photosynthesis analysis, SOD = superoxide dismutase, POD = peroxidase, APX = ascorbate peroxidase, CAT = catalase, Si% (leaf) = silicon percentage in leaves, Si% (root) = silicon percentage in roots, RDW (g/plant) MT = root dry weight at maximum tillering stage, RDW (g/plant) PI = root dry weight at panicle initiation stage, RDW (g/plant) = root dry weight at flowering stage, SDW (g/plant) MT = shoot dry weight at maximum tillering stage, SDW (g/plant) PI = shoot dry weight at panicle initiation stage, and SDW (g/plant) = shoot dry weight at flowering stage.
